# Reproductive mode and fine-scale population genetic structure of grape phylloxera (*Daktulosphaira vitifoliae*) in a viticultural area in California

**DOI:** 10.1186/1471-2156-14-123

**Published:** 2013-12-24

**Authors:** Md Sajedul Islam, Tamara L Roush, Michael Andrew Walker, Jeffrey Granett, Hong Lin

**Affiliations:** 1 Department of Viticulture and Enology, University of California Davis, One Shields Avenue, Davis, CA 95616, USA; 2 Department of Entomology, University of California Davis, One Shields Avenue, Davis, CA 95616, USA; 3 USDA, Agricultural Resarch Service, USDA-ARS, San Joaquin Valley Agricultural Sciences Center, 9611 South Riverbend Avenue, Parlier, CA 93648-9757, USA

**Keywords:** *Daktulosphaira vitifoliae*, Grape phylloxera, Microsatellite marker, Genetic diversity, Genetic structure, Reproductive mode

## Abstract

**Background:**

Grape phylloxera (*Daktulosphaira vitifoliae*) is one of the world’s most important viticultural pests. However, the reproductive mode, genetic structure and host adaptation of phylloxera in various viticultural environments remains unclear. We examined reproductive mode and genetic structure of phylloxera by analyzing microsatellite makers across the samples from four vineyard-sites in California.

**Result:**

The phylloxera populations in California are believed to have predominantly parthenogenetic reproduction. Therefore, genetic diversity of phylloxera is expected to be limited. However, this study showed relatively high levels of diversity in Napa and Yolo county populations with a large number of unique genotypes, average number of alleles (2.1 to 2.9) and observed heterozygosities (0.330 to 0.388) per vineyard-sites. Reproduction diversity index (G: N—unique genotypes versus number of samples) ranged from 0.500 to 0.656 among vineyard-sites. Both significant and non-significant *P*_sex_ (probability of sexual reproduction) were observed among different repeated genotypes within each vineyard. Moreover, high variation of *F*_IS_ was observed among different loci in each vineyard-site. Genetic structure analysis (UPGMA) and various measures of population differentiations (*F*_ST_, PCA, and gene flow estimates) consistently separated AXR#1 (*Vitis vinifera* x *V. rupestris*—widely planted in California during the 1960s and 1970s) associated populations from the populations associated with other different rootstocks.

**Conclusion:**

Genetic diversity, G: N ratio, *P*_sex_ and *F*_IS_ consistently suggested the occurrence of both parthenogenetic and sexual reproduction in California populations. This study clearly identified two major groups of phylloxera obtained from various rootstocks, with one group exclusively associated with only AXR#1 rootstock, defined as “biotype B”, and another group associated with vinifera-based rootstocks, known as “biotype A”.

## Background

Grape phylloxera (*Daktulosphaira vitifoliae*) Fitch (Hemiptera: Phylloxeridae) is an economically important pest that specializes in feeding on grapevine (*Vitis* spp.). It is an aphid-like pest that forms pocket like galls on leaves and hooked galls (nodosities) on the young root tips. This pest has destroyed vineyards around the world for the past 150 years and is regarded as one of the world’s most important viticultural pests [1].

Phylloxera are found throughout the Americas where they appear to have coevolved with the endemic *Vitis* spp [2], which have varying levels of tolerance or resistance to it. The use of rootstocks, developed in Europe from resistant North American grape species, has proven to be an effective means of controlling phylloxera for more than 100 years in Europe, California and around the world as phylloxera spread. Although these resistant rootstocks resist feeding on storage roots (tuberosities), they do allow varying degrees of feeding and nodosity development on their feeder roots. Rootstocks with partial *V. vinifera* parentage can allow damaging tuberosities to develop. The hybrid rootstock AXR#1 (*V. vinifera* x *V. rupestris*), which was widely planted in California during the 1960s and 1970s, succumbed to an outbreak of adapted phylloxera strains in the 1980s. Based on biological and behavioral characteristics these AXR#1 damaging strains were previously defined as biotype B to distinguish them from strains that could damage *V. vinifera* grapevines but not AXR#1, which were named biotype A [3-5]. It is still unknown whether biotype B strains were selected by the use of AXR#1 rootstock, whether they were imported from other regions, or were derived from existing strains.

The life cycle and mode of reproduction of phylloxera from various viticultural environments in the world still remains a subject of discussion and confusion [6]. Like other Aphidoidea, phylloxera are thought to have a holocyclic life cycle with alternating phases of sexual and asexual reproduction [7]. These phases include parthenogenetic generations on the roots or leaves and the possible occurrence of a sexual phase that may link the asexual root and leaf forms. While the “classical” description of the life cycle is regarded as holocyclic or cyclic parthenogenesis (alternating between asexual and sexual life phases on the same host), anholocyclic (asexual) reproduction and parthenogenetic lineages are predominantly reported for grape phylloxera in various grape growing environment including Australia (northeast and central Victoria) and parts of Europe [8-10]. However, holocyclic (sexual) reproduction was also inferred in European vineyard [8,11]. In fact, the life cycle, reproductive mode and population structure of phylloxera may vary depending on the genetic characteristics of the insect, its *Vitis* hosts and environment conditions, leading to strains that feed on roots, leaves, and in some cases both grapevine tissues.

Phylloxera in California are present mainly on the root system and are thought to be functionally parthenogenetic due to the rarity of leaf galls and the observation that juvenile hibenants can overwinter on the root system [7]. A molecular study with limited numbers of samples also inferred that parthenogenesis is perhaps the primary reproductive mode in California [12]. Whether sexual reproduction occurs in California and the degree to which it exists elsewhere is largely unclear, but needs to be investigated for a better understanding of the genetic diversity and population structure of phylloxera, and so that control measures can be developed. Information about the reproductive characteristics and fine-scale population genetic structure of phylloxera is important for understanding the evolutionary potential for this pest to adapt to resistant rootstocks, and how it colonizes and migrates among vineyards. This information might also shed light on the origin and distribution of different strains among various vineyards in a small-scale geography.

The use of molecular markers to examine the extent of genetic variation in an agricultural system can provide insights into pest population dynamics over time and space. DNA-based molecular markers have been used to evaluate the reproductive mode and genetic variation of phylloxera in various viticultural areas in the world. Forneck et al. [11] characterized European populations using AFLPs and suggested that there were two independent origins of phylloxera into European vineyards, and that genetic structure was not associated with hosts. Phylogenetic analyses of mtDNA sequences have shown that two divergent grape phylloxera lineages were introduced into global viticulture [13]. In addition, highly variable microsatellite makers have been used to facilitate the assessment of the reproductive mode, and to evaluate genetic structure of various Australian and European populations [8-10]. These studies suggested that reproduction was predominantly asexual, that host associated asexual lineages existed, and that populations can differ between leaf and root forms. However, the precision with which California phylloxera have been examined has lagged behind these efforts. RAPD markers were mostly used to examine the genetic diversity of California phylloxera [14], and United States populations [15,16]. Later, a small numbers of California samples were evaluated with microsatellites markers [12]. However, relatively limited information was obtained from the RAPD study and the small sample size impacted conclusions from the latter study.

Microsatellite markers can provide a powerful system for unraveling life history traits, particularly the occurrence of sexual reproduction [17,18]. This marker system has been used effectively in life cycle studies of members of the Aphididae family, resulting in relatively high levels of resolution for determination of reproductive mode and genetic relationships [19-22]. However, development of microsatellite markers has proved difficult in grape phylloxera [9]. Initially, only a few such markers were developed, but they proved useful for studying phylloxera populations in Australia and Europe [8-10]. Additional microsatellite markers were developed later in a separate study, and were characterized using samples from Europe and California [12]. In this study, we incorporated multilocus microsatellite markers from these previous studies [9,12] to analyze phylloxera populations recovered from four different vineyard-sites within two adjacent counties, Napa County (Oakville) and Yolo County (Woodland) in California. The objectives of this study were: i) to understand the reproductive mode of phylloxera in California viticulture; and ii) to analyze genetic diversity, gene flow and genetic population structure of this pest among various vineyards in a small-scale geography.

## Results

### Genotypes and reproduction

We analyzed 225 phylloxera samples from four fine-scale populations obtained from four different vineyard sites from Oakville of Napa and Woodland of Yolo counties in California (Table 1). In total 106 genotypes were identified across the overall samples based on the combination of allelic data from eight microsatellite markers. Multilocus repeated genotypes (genotype observed more than once in a population) were observed within each of the study sites. The distribution of genotype classes among the different sites are reported in Additional file 1: Table S1. Reproduction diversity (G: N ratio) ranged from 0.500 to 0.656 among the populations (Table 2). The probability of an independently produced repeated genotype in a population by sexual reproduction (without clonal reproduction) as determined by level of significances of *P*_sex_ values from MLGsim simulations is presented in Table 2. The significant and non-significant *P*_sex_ values among different repeated genotypes within each population suggested both clonally and sexually reproduced repeated genotypes. However, the proportions of the sexually or asexually reproduced repeated genotypes varied from population to population. A relatively larger proportion of clonally reproduced repeated (significant *P*_sex_) genotype sets was observed in UCD-OKV (10 out of 13) and Col-2 (4 out of 7) populations than Co1-1 (2 out of 6) and Woodland (1 out of 5) populations. Sexually produced repeated genotypes were relatively higher in Co1-1 and in the Woodland populations.

**Table 1:** Sample information of grape phylloxera from four vineyard-sites in Napa (Oakville) and Yolo (Woodland) counties, California

**Population ID**	**Sample locations**	**Rootstock host**^**1**^	**Number of individuals**
Col-1	Collins-Block-1, Oakville, Napa	AXR#1	52
Col-2	Collins-Block-2, Oakville, Napa	5C	63
UCD-OKV	University of California Davis (UCD) Oakville Station, Napa	5C, 101-14Mgt and 1103P	78
Woodland	Woodland, Yolo	110R and 101-14Mgt	32

^1^, AXR#1 = *V. vinifera* x *V. rupestris*; 5C = *V. berlandieri* x *V. riparia*; 101-14Mgt = *V. riparia* x *V. rupestris*; 1103P = *V. berlandieri* x *V. rupestris*; 110R = *V. berlandieri* x *V. rupestris.*

**Table 2:** Genetic diversity parameters of grape phylloxera populations across four vineyard-sites in Napa (Oakville) and Yolo (Woodland) counties, California

**Population ID**	**Number of individuals**	**Number of distinct genotypes**	**G/N**	**Number of repeated genotypes**	**Number of significant *P***_**sex**_	**Number of non-significant *P***_**sex**_	**Mean alleles**	**Mean *H***_**o**_	**Mean *H***_**E**_	***P *** **(HWE)**	***F***_**IS **_**multilocus**^**-MCG**^
Col-1	52	30	0.577	6	2	4	2.1	0.388	0.326	***	-0.190
Col-2	63	35	0.555	7	4	3	2.8	0.339	0.357	***	0.050
UCD-OKV	78	39	0.500	13	10	3	2.8	0.330	0.398	***	0.170
Woodland	32	21	0.656	5	1	4	2.9	0.375	0.368	0.137	-0.020

G/N, Genotypic (reproduction) diversity index, *P*_sex_, Significantly clonal genotypes, *H*_O_, Observed heterozygosity, *H*_E_, Expected heterozygosity, *P* (HWE), Exact *P* values for Hardy-Weinberg equilibrium test, *F*_IS_ Multilocus^-MCG^, *F*_IS_ averaged over loci (calculations without multicopy genotypes), ***, *P* < 0.001 (highly significant).

Hardy-Weinberg exact probability tests showed significant deviation from expectations for the populations from three sites (Col-1, Col-2, and UCD-OKV) across the tested loci (Table 2). However, locus-wise analysis showed that some loci did not significantly deviate from Hardy-Weinberg equilibrium (HWE) within these populations (Table 3). While the Woodland population did not significantly deviate from Hardy-Weinberg equilibrium (HWE) expectations across all loci, three loci showed HWE deviation in this population (Table 3). The *F*_IS_ value across the overall loci was significant in the UCD-OKV population. This value was low in Col-2. The negative *F*_IS_ across overall loci in Col-1 and Woodland populations indicates that they had a higher portion of heterozygotes than the UCD-OKV and Col-2 populations (Table 2). Locus-wise comparisons showed that there were high variations among *F*_IS_ values within each of the populations, where the distributions of both positive and negative *F*_IS_ were observed at various loci (Table 3). In the clonal-corrected dataset, six of the eight microsatellite loci showed linkage disequilibrium when paired with each other (*P* < 0.05).

**Table 3:** Genetic diversity estimates at eight microsatellite loci across the grape phylloxera populations from four vineyard-sites in Napa (Oakville) and Yolo (Woodland) counties, California

**Population ID**	**Locus**	**N**	**Na**	**Ne**	***H***_**O**_	***H***_**E**_	***F***_**IS**_	***P *** **(HWE** ***)***
Col-1	DVIT1	30	2.0	2.0	1.000	0.500	-1.000	*
	DVIT2	30	3.0	2.3	0.633	0.572	-0.108	0.136
	DVIT3	30	2.0	1.8	0.633	0.433	-0.463	*
	DVSSR4	30	3.0	1.6	0.433	0.365	-0.187	*
	DVSSR6	30	1.0	1.0	0.000	0.000	N/A	-
	DVSSR7	30	2.0	1.3	0.233	0.206	-0.132	1.000
	DVSSR16	30	1.0	1.0	0.000	0.000	N/A	-
	DVSSR17	30	3.0	2.1	0.167	0.529	0.685	*
Col-2	DVIT1	35	5.0	2.6	0.800	0.619	-0.292	*
	DVIT2	35	2.0	1.8	0.343	0.431	0.205	*
	DVIT3	35	3.0	1.2	0.171	0.159	-0.080	1.000
	DVSSR4	35	3.0	2.4	0.629	0.579	-0.085	1.000
	DVSSR6	35	2.0	1.1	0.000	0.108	1.000	*
	DVSSR7	35	2.0	1.1	0.000	0.056	1.000	*
	DVSSR16	35	2.0	1.6	0.457	0.382	-0.197	0.402
	DVSSR17	35	3.0	2.1	0.314	0.524	0.400	*
UCD-OKV	DVIT1	39	6.0	2.7	0.718	0.625	-0.149	*
	DVIT2	39	3.0	2.2	0.385	0.536	0.283	*
	DVIT3	39	2.0	1.1	0.103	0.097	-0.054	1.00
	DVSSR4	39	3.0	2.9	0.744	0.651	-0.143	*
	DVSSR6	39	3.0	1.4	0.051	0.308	0.833	*
	DVSSR7	39	1.0	1.0	0.000	0.000	N/A	-
	DVSSR16	39	2.0	1.9	0.282	0.467	0.396	*
	DVSSR17	39	2.0	2.0	0.359	0.500	0.282	*
Woodland	DVIT1	21	4.0	2.7	0.714	0.625	-0.143	*
	DVIT2	21	4.0	2.2	0.524	0.545	0.040	1.000
	DVIT3	21	3.0	1.8	0.571	0.459	-0.244	*
	DVSSR4	21	4.0	2.4	0.571	0.577	0.010	0.371
	DVSSR6	21	2.0	1.0	0.048	0.046	-0.024	1.00
	DVSSR7	21	1.0	1.0	0.000	0.000	N/A	-
	DVSSR16	21	2.0	1.2	0.095	0.172	0.447	*
	DVSSR17	21	3.0	2.1	0.476	0.516	0.077	0.530

N, Number of samples; Na, Number of alleles; Ne, Number of effective alleles; *H*_O_, Observed heterozygosity; *H*_E_, Expected heterozygosity, *F*_IS_, Fixation index;*, *P* < 0.05 (significant).

### Genetic diversity

The average number of alleles per locus per population ranged from 2.1 (Col-1) to 2.9 (Woodland) (Table 2). Locus-wise allelic diversity, observed and expected hererozgosities for each population are presented in Table 3. Similar levels of genetic diversity were observed at each of the populations. Observed hererozygosities across all eight microsatellite loci among the populations ranged from 0.388 (Col-1) to 0.330 (UCD-OKV) (Table 2). Comparatively higher numbers of distinct alleles (at 3 loci) were found within Col-1.

### Genetic structure

Genotypic classes within the Col-1 site (planted with AXR#1) were completely distinct and did not overlap with the samples from other sites, including the adjacent Col-2 site. The frequency of unique alleles was also relatively high in Col-1 (at three loci). While a large number of distinct genotypes were also found at each of the other three study sites, several repeated genotype classes were observed to overlap among these sites planted with varieties of rootstocks. For example, four genotypes (G35, G38, G62 and G9; highlighted with gray color in Additional file 1:Table S1) were distributed among these three sites, containing various types of rootstocks including 5C, 1103P, 110R, and 101-14Mgt. The frequency of sharing repeated genotypes; however, was higher between two Oakville populations (Col-2 and UCD-OKV) than when Woodland was compared to these Oakville populations.

*F*_ST_ values indicated very high differentiation and distinct genetic structure between Col-1 and each of the populations from other sites (*F*_ST_, 0.398 to 0.431). If the Col-1 population was excluded, differentiation was low in comparisons among the other three populations (*F*_ST_, 0.015 to 0.105). Two Oakville (Col-2 and UCD-OKV) populations had the lowest differentiation (*F*_ST_, 0.015). However, the differentiations were relatively higher between each of the Col-2 and UCD-OKV populations and the Woodland population (Table 4).

**Table 4:** **Genetic differentiation (*F***_**ST**_**) among the populations of grape phylloxera from four vineyard-sites in Napa (Oakville) and Yolo (Woodland) counties, California**

**Population ID**	**Col-1**	**Col-2**	**UCD-OKV**
Col-2	0.431		
UCD-OKV	0.408	0.015^NS^	
Woodland	0.398	0.105	0.073

^NS^, Non-significant genetic differentiations (*P* > 0.01).

**Figure 1:**
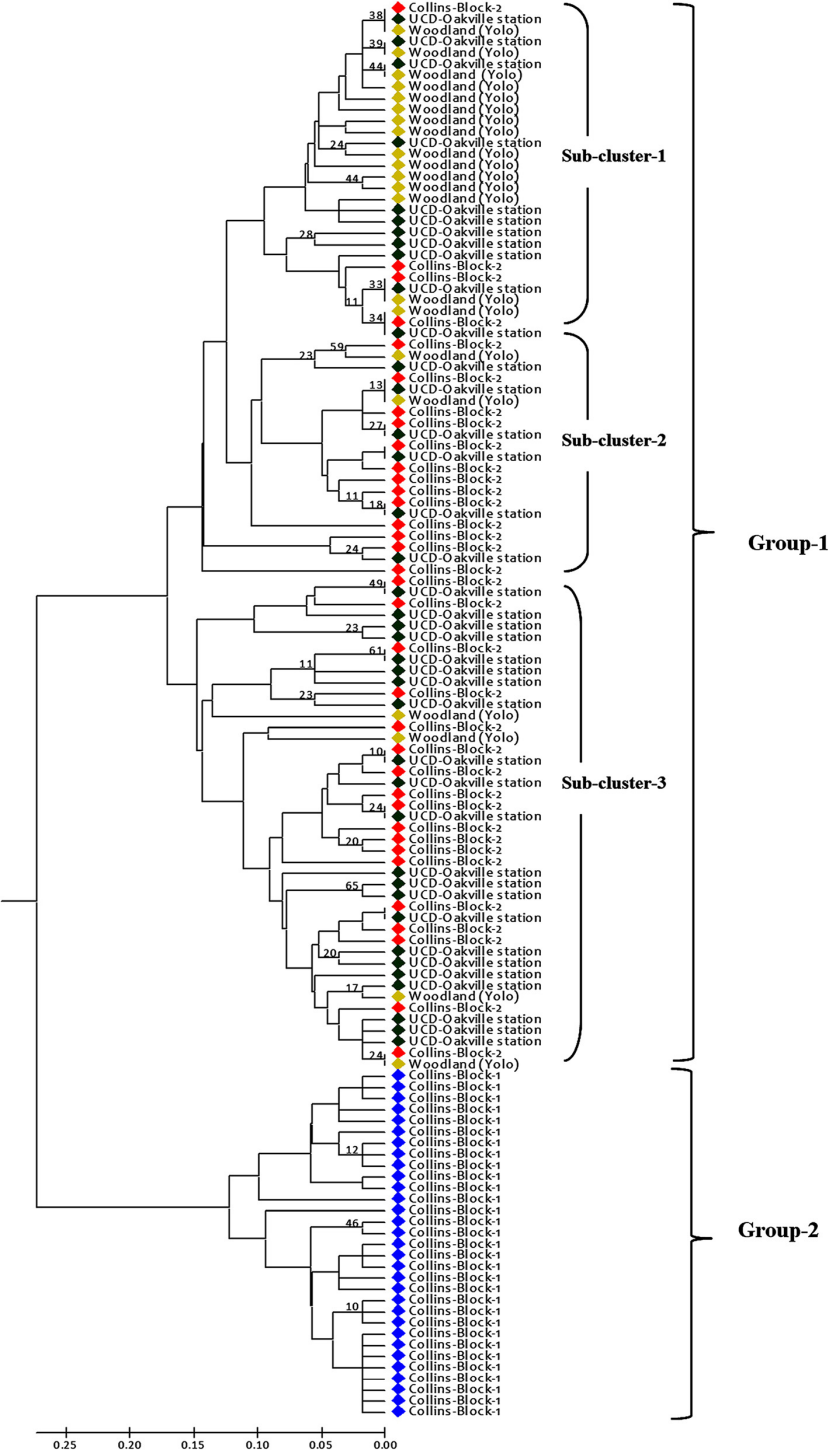
**Genetic relationship among grape phylloxera samples from four vineyard**- **sites in Napa (Oakville) and Yolo (Woodland) counties, California, as revealed by UPGMA clustering analysis.** Clonal corrected data were used and the dendrogram was constructed by computing distances between individual samples based on the DA distance [46]. Only bootstrap values >25% are shown

UPGMA clustering analysis further evaluated the genetic relationship and structure of phylloxera samples across the samples. Broadly two large groups were detected: one group with samples from UCD-OKV, Col-2 and Woodland; and the other from Col-1 (Figure 1). While all samples from Col-1 were within the second group, apparently three sub-clusters were observed among the samples obtained from the other sites, where samples from Col-2 and UCD-OKV were somewhat distributed among all three sub-clusters, but they were found less often in the sub-cluster-1(Figure 1). A small number of samples from Woodland were included in sub-cluster-2 and 3, but most Woodland samples were included in sub-cluster-1.

**Figure 2:**
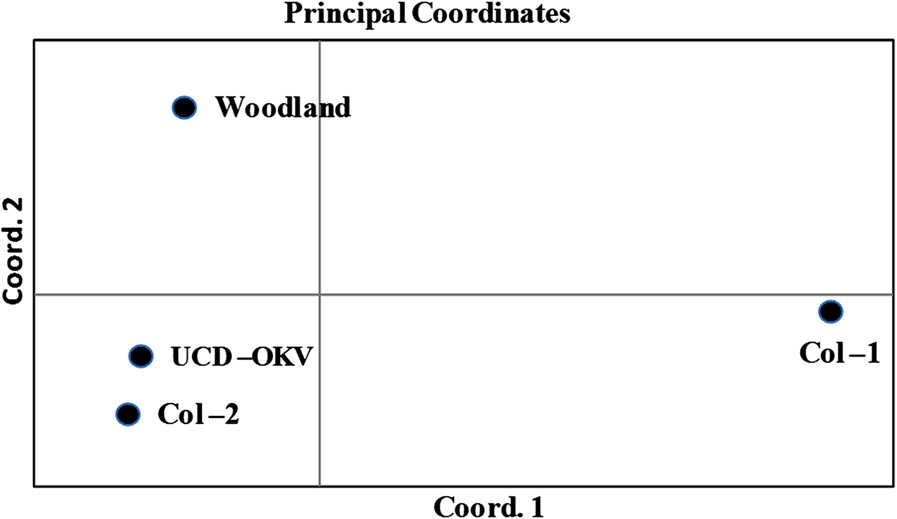
**Plot of the principal coordinate analysis (PCA) from the covariance matrix with data standardization calculated using GenAlEx for the grape phylloxera populations from four vineyard**-**sites in Napa (Oakville) and Yolo (Woodland) counties, California.**

Finally, a PCA analysis evaluated the overall pattern of variation among the populations with a graphical representation. The PCA chart shows that the Col-1 appeared in the middle-end of the right hand quadrant; clearly separated from the other three populations that all appeared in the left hand quadrant. Furthermore, genetic structure was observed between Woodland and each of the Col-2 and UCD-OKV population, where Col-2 and USD OKV clustered together on lower part of the left hand quadrant, and Woodland appeared in the upper part of left and quadrant (Figure 2).

### Gene flow

Gene flow (*m*) among the populations was estimated to determine if these values were consistent with measures of population structure. Estimates and direction of gene flow from BayesAss analysis are presented in Table 5. BayesAss indicated that gene flow between Col-1 and each of other three populations was undetectable from those generated by uninformative data, which lack sufficient variation to detect dispersal events with high confidence. Analysis showed that *m* values less than 0.055 are indistinguishable from those generated by uninformative data [23]. However, gene flow among the other three populations showed “measurable” dispersal rates given our genotypic data. Generally, a similar level of gene flow was found among these populations. However, a slightly higher rate of dispersal was observed between Col-2 and UCD-OKV Oakville populations from both directions, and a relatively low rate of gene flow was observed between Woodland and each of the Oakville populations (Table 5).

**Table 5:** Rate of gene flow estimates (both direction), inferred from genetic assignment from BayesAss analysis, among the grape phylloxera populations from four vineyard-sites in Napa (Oakville) and Yolo (Woodland) counties, California

**Population ID**	**Col-1**	**Col-2**	**UCD-OKV**	**Woodland**
Col-1		0.048 (0.000-0.201)	0.048 (0.000-0.197)	0.046 (0.000-0.199)
Col-2	0.049 (0.000-0.186)		0.061 (0.000-0.231)	0.068 (0.000-0.240)
UCD-OKV	0.052 (0.000-0.193)	0.070 (0.000-0.239)		0.076 (0.000-0.247)
Woodland	0.045 (0.000-0.177)	0.062 (0.000-0.236)	0.063 (0.000-0.220)	

Numbers in parentheses represent 95% confidence intervals. The direction of gene flow is from the population in the left column of the table to the population along the top row.

## Discussion

### Reproductive characteristics

Studies on the life cycle and reproductive mode of phylloxera have been of considerable scientific interest and importance for viticulture since its emergence as a key viticultural pest about 150 years ago. However, the life cycle and reproductive mode of phylloxera remains a subject of discussion and confusion [6]. Phylloxera are traditionally thought to have a holocyclic life cycle with alternating sexual and asexual reproductive phases [7]. Given these reproductive characteristics, repeated genotypes observed in our microsatellite analysis are, therefore, as expected within each of the study sites. The repeated genotypes could result from clonal reproduction; however, other reproductive systems such as sexual reproduction can lead to the occurrences of repeated genotypes in highly subdivided populations [24]. MLGsim analysis suggested both clonally and sexually reproduced repeated genotypes within each of the populations. However, the relatively large proportion of sexually produced (non-significant *P*_sex_) repeated genotypes, especially in the Woodland population, might be attributed to the establishment of leaf gall population on the *Vitis* hybrid rootstocks.

Asexual reproduction tends to decrease segregation of alleles within loci and recombination between loci. Over time, this leads to observed heterozygosities (*H*_O_) differing from those expected under sexual outbreeding (*H*_E_), and deviations from HWE as described by Ivens et al. [25]. The significant deviations from HWE Co1-1, Co1-2, and UCD-OKV populations across all loci, and across some of the loci in the Woodland population are, therefore, most likely attributed from the asexual reproduction. Negative *F*_IS_ values across overall loci at Col-1 and the Woodland populations are, therefore, due to pervasive clonal reproduction relative to random mating. While *F*_IS_ was positive at Co1-2 and UCD-OKV populations across all loci, asexual reproduction was expectedly suggested in these populations as documented by the negative *F*_IS_ at some of the loci (Table 3) as well from the deviation of HWE across the overall loci. Finally, significant linkage disequilibrium among the pairs of six microsatellite loci across the overall populations might have resulted from the lack of recombination under asexual reproduction. Nevertheless, high variation of *F*_IS_ values (from positive to negative) among various loci in each of the study populations and non-significant HWE at the corresponding loci in these populations suggests that sexual recombination events do occur on some points [26]. The appearance of a large number of unique genotypes in these populations also supports the existence of sexual reproduction [26,27] or establishment populations from sexually reproduced individuals.

It has been reported that parthenogenesis is the dominant mode of reproduction for phylloxera in California, and that California populations are apparently only anholocyclic, or largely asexually [1,3,7,9,12,15]. This assumption is most likely based on negative evidence such as unobserved males and the absence of leaf galls, which are assumed to be initiated by sexually produced overwintering eggs. However, microsatellite analysis in the present study suggests that in addition to parthenogenesis, sexual recombination also occurred to a greater or lesser extent at each of the sites studied.

It is assumed that phylloxera populations have a holocyclic life cycle (with sexual reproduction) in their native range, whereas an anholocyclic one (completely parthenogenetic) is thought to be more common in the introduced range. [13]. However, evidence of sexual recombination events along with predominant anholocyclic (asexual) reproduction was reported in various parts of Europe from molecular [8,11] as well as from a classical life cycle study [28]. Moreover, the occurrence of sexual reproduction, along with predominant asexual reproduction, was not dismissed in Australian vineyards, where few of the sexually generated and statistically expected genotypes were found [9,10]. In fact, the life cycle of grape phylloxera is not fixed and populations can adapt to specific habitat conditions and grape species hosts, which may influence their reproductive behavior [29]. While multiple introductions from various founders derived from sexually reproducing populations may have had some influence on the observed genetic diversity and measures of reproductive characteristics within California phylloxera populations, it is likely that sexual reproduction exists at some level in established populations.

### Genetic diversity within populations

Given the parthenogenetic life cycle of phylloxera in California [7], limited genetic diversity is expected. However, moderately high levels of genetic diversity within phylloxera populations were detected based on the average numbers of alleles and observed level of heterozygosities in this study. The first study of phylloxera genetic diversity in California was done using RAPD markers and it found relatively high levels of polymorphism given that few differences in phylloxera feeding behavior had been detected [14]. High levels of diversity were also found when collections from other parts of the United States were studied with RAPD markers [16]. Sequence variation of mitochondrial DNA detected variable levels of genetic variation in native and agricultural populations of phylloxera across the United States [13].

The likely cause of what was assumed to be high genetic diversity in a parthenogenetic insect was multiple introductions [3]. Davidson and Nougaret [7] in an early study of California phylloxera considered that they had been imported from the eastern United States. Downie [13] analyzed mitochondrial genes to describe the origin of California phylloxera and found the majority of sampled haplotypes to be similar to strains collected from the eastern United States on *V. vulpina* (a species common in the southeastern and central United States). He also found that the California strains were genetically distinct from strains of European phylloxera.

New clonally based genetic diversity is expected if an introduced genotype successfully adapts to a new environment. Increases or decreases in a population’s genetic diversity also depend on the rate of mutation and the fitness of new genotypes in a population. Mutation can also be neutral if mutated loci are not subject to selection pressure. Phylloxera is considered to have been introduced into California about 150 years ago, however, the mutation rate of the grape phylloxera genome and its contribution to population diversity in California vineyards is not clear [30]. An undetected sexual phase of the life cycle may be a key factor contributing to high genetic diversity within population at different vineyard-sites. If the genotypes were the result of recombination followed by expansion of lineages by parthenogenesis, the majority of samples of any one genotypic class should be found within the same location and in the presence of related recombinant genotypes [9], since phylloxera has limited capacity for dispersal given its small size, and the fact that the flying forms of this insect do not feed [10]. The large numbers of distinct genotypes with repeated genotype classes in every population observed in our analysis in California vineyards are, therefore, likely to have resulted from sexual reproduction followed by expansion of lineages by parthenogenesis.

### Genetic structure and gene flow

Microsatellite analysis presented here revealed a distinctive genetic structure of the Col-1 population collected from AXR#1 rootstock. While morphological traits that distinguish biotype B from other strains have not been detected, the genetic structure of the biotype B strains observed in this study is consistent with the biological and behavioral characteristics of these AXR#1 feeding types. It is likely that the biotype B population found at the Col-1 site was introduced or evolved in place, and then developed into a genetically unique colony. Our results suggest that the Col-1 biotype B strains did not give rise to the different strains found in the adjacent vineyard Col-2, where AXR#1 was pulled out in the early 1990s and 5C was used to replant the vineyard. Rather, Col-2 strains were imported on plant material or roots from other vineyards or they also evolved in place. By definition biotype B reproduces quickly and causes decline of AXR#1 rootstock. It caused large-scale replanting of California vineyards, which have been replaced with a wide range of phylloxera resistant rootstocks. Type B populations may be still survive in some vineyards and may be slowly adapting to alternative rootstock hosts.

Host plants have been reported to be an important factor influencing adaptation of races or demes in aphids [31]. Corrie et al. [9] and Corrie and Hoffmann [10] reported strong associations between asexual lineages and host types in vineyards. Excised root bioassays [32] and an aseptic dual culture system [33] demonstrated that phylloxera can readily form host-adapted strains. Corrie, et al. [34] also reported strong associations between a grape host genotype and the asexual lineages. However, sampling from various viticultural regions throughout Europe did not find a host association [11]. In another analysis, native grape phylloxera on two sympatric host species did not cluster [35]. Host association lineages were also not observed in China [36]. Thus, both host-associated and non-host-associated populations of phylloxera could be established in various viticulture environments depending on the biotype, selectively adaptive advantages with favorable ecological or biological conditions or on the time frame required for a strain to adapt to particular host in a particular viticultural environment.

Our study found host-associated genetic structure with the biotype B phylloxera at the Col-1 site, but did not show host associations among the other stains and rootstock hosts including 5C, 1103P, 110R, and 101-14Mgt. For example, when Col-1 data was excluded, much lower levels of differentiation and similar levels of gene flow were observed among the remaining sites containing the other rootstocks. Differentiation was also significantly lower between the two closely located Oakville sites (Col-2 and UCD-OKV). The Woodland population was, however, reasonably differentiated from two of the Oakville (Col-2 and UCD-OKV) populations in terms of genetic differentiation and the level of gene flow. Given phylloxera’s limited dispersal ability and the long distances between the sites, the lower rates of gene flow and relatively high differentiation between the Woodland and the two Oakville populations were expected. Moreover, the higher level of genetic differentiation between the two Oakville populations (obtained from roots) and the Woodland population (obtained from leaf galls) than when the two Oakville populations were compared to each other could have resulted from the different genetic composition of phylloxera populations inhabiting root and leaf galls, respectively. Differences of lineages from root and leaf gall samples were observed in Australian Vineyards [9].

## Conclusion

Our analysis suggested both parthenogenetic and sexual reproductive modes in phylloxera exist in California. Various measurements of population differentiations at microsatellite loci clearly identified two major genetic groups, with one group associated with AXR#1, and another group associated with non-AXR#1 rootstock. While host-associated genetic structure was not observed within other strains and populations, a moderate differentiation was observed among the populations based on spatial distance, or based on the population inhabiting grapevine roots and leaves. While our results here provide some insights into the genetic diversity, reproductive mode and genetic structure of grape phylloxera in California, it should be noted that our sampling is certainly not all inclusive; broader population analysis from phylloxera’s wide geographical distribution will be needed for further resolution.

## Methods

### Sampling details

Four study sites were selected from vineyards in two adjacent counties in California: Napa and Yolo. Samples were collected from three sites at Napa [(1) Collins-Block-1 (Oakville); (2) Collins-Block-2 (Oakville); (3) the University of California Davis (UCD) Oakville station] and one site at Yolo County (Woodland). Detailed sample information and population IDs are presented in Table 1. Phylloxera samples were randomly collected from these study sites regardless of their association with any specific rootstocks.

The Collins site is a 6 hectare vineyard in Oakville, Napa County, CA that was originally planted with AXR#1 (*V. vinifera* x *V. rupestris*) rootstock. The south end of the Collins site (about a 2 hectare block; Col-1) was still planted with the original AXR#1 at the time of sampling. However, the adjoining 4 hectares of the Collins site (Col-2) were replanted with the rootstock 5C (*V. berlandieri* x *V. riparia*) after the AXR#1 failed to phylloxera. Samples from the University of California, Davis Oakville Experimental Station (UCD-OKV) were collected from about a 1 hectare block planted with the rootstocks 5C, 1103P and 101-14Mgt. This site was also replanted over an AXR#1 block that failed to phylloxera. The Woodland samples were collected from leaf galls that had formed at a rootstock nursery block containing 110R and 101-14Mgt.

Infested roots were dug from three study sties in Napa and placed in separate plastic bags along with some soil for transport to the laboratory. Healthy, lignified roots 2-6 mm in diameter were also cut from each vine. Plastic bags containing infested and healthy roots were kept at room temperature until enough eggs were produced for DNA extraction. Samples from Woodland population were taken from foliar phylloxera galls. Eggs were removed from multiple galls per sampled vine and they were pooled for DNA isolation.

### DNA extraction

Approximately 50-200 phylloxera eggs from each collection were transferred to microcentrifuge tubes with fitted microgrinders (Radnoti Glass, Arcadia, CA) for DNA extraction as described by [37]. DNA concentrations were calculated from measurements at OD_260_ and adjusted to 10 ng/μl with molecular grade water.

### PCR amplification and fragment analysis

Eight microsatellite markers from phylloxera were employed in the present study. Five of these markers (DVSSR4, DVSSR6, DVSSR7, DVSSR16, and DVSSR17) were described in [12] and three (DVIT1, DVIT2, DVIT3) were described in [9]. The forward primer of each pair was labeled with a fluorescent dye (Applied Biosystems, Foster City, CA). Each 20 μl PCR reaction contained 1× reaction buffer, 1.5 mM MgCl_2_, 1U *Taq* polymerase (Applied Biosystems) 0.2 mM dNTP (Applied Biosystems), 0.25 pM of the labeled primer, 0.25 pM of the unlabeled primer and 20 ng DNA. A PTC-100 (MJ Research Inc., USA) was used to run the reactions with the following program: 95°C for 5 min followed by a 40 cycles of 95°C for 30 sec, 58°C for 30 sec and 72°C for 1 min, and a final extension at 72°C for 10 min.

Then 1 μl of PCR product was added to 10 μl deionized formamide and 0.15 μl of molecular size standard (GENESCAN 500 ROX). The mixed PCR products were then loaded on to an ABI PRISM 3100 Genetic Analyser (Applied Biosystems) with 36-cm capillaries filled with polymer POP-6 module. The data were analyzed by GeneMap 4.0 (Applied Biosystems).

### Genotyping and analysis of reproductive characteristics

Allelic data obtained from multilocus microsatellite markers were combined and genotypes were identified. Several sets of repeated genotypes were identified within the population at each study site. The probability of observing *n* times a multilocus genotype in a population and the likelihood of them having resulted from clonal reproduction was tested using MLGsim software [38]. Based on the observed allele frequencies, *P*_sex_ values were calculated for every set of repeated genotypes at each population as suggested by Halkett et al. [24]. Using a Monte Carlo simulation method, the MLGsim determines the significance threshold for *P*_sex_ values, identifying repeated copy multilocus genotypes that did not occur by chance from sexual reproduction (true clones). Calculations were done for each population, taking into account sample size and allele frequencies. The significance level was set to 0.05.

To estimate reproduction diversity, a diversity index was calculated for each population using the G: N ratio, where G is the total number of unique genotypes found across all samples and N is total number of samples. The G:N ratio ranges from 0– all individuals share the same genotype, in case of strict clonality to 1– all individuals have distinct genotypes, under sexual reproduction [39].

### Genetic diversity analysis

Under the very likely condition that repeated (clonal) ‘amplification’ is not equal over all genotypes, unwitting inclusion of clonal copies in population genetic analyses has the potential to mislead [17]. Therefore, to prevent distorted estimates for heterozygosity and *F-*statistics due to the presence of identical copies of clonal genotypes in the populations tested, a clonal-corrected (a single copy from each set of identical genotypes) data set was built, and applied to the analyses.

GenAlEx Version 6.3 [40] was used to calculate number of alleles, observed heterozygosity (*H*_O_), Nei’s unbiased expected heterozygosity (*H*_E_) [41] per microsatellite locus per population. FSTAT Version 2.9.3.2 [42] was used to calculate Wright’s inbreeding coefficient (*F*_IS_) [43] within each population. Significance of the deviation of *F*_IS_ from zero within population was determined using the FSTAT randomization test. Hardy–Weinberg equilibria over all loci and populations were tested using GENEPOP web version 4.0.10 [44]. A Markov chain (MC) algorithm was used to estimate exact P-value of this test [45]. GENEPOP were also used to test linkage disequilibrium between each pair of microsatellite loci.

### Analysis of genetic structure

GENEPOP web version 4.0.10 [44] was used to calculate pairwise *F*_ST_ as a basic matrix of population genetic structure and differentiation. A significance test of *F*_ST_ was performed using FSTAT ver. 2.9.3.2 [45], where the levels of significance were adjusted for multiple tests according to the Bonferroni corrections. To understand the genetic structure of grape phylloxera, a UPGMA dendrogram was also constructed based on Nei’s DA genetic distance [46] between individual samples collected from four of the study sites. Trees were constructed using the POPULATION software package version 1.2.31 (Olivier Langella, CNRS UPR9034, France http://bioinformatics.org/~tryphon/populations) and graphically displayed with MEGA4 software [47]. Confidence in specific clusters of the resulting topology was estimated by bootstrap analysis with 1,000 replicates.

Finally, to evaluate the overall patterns of population variation within a multivariate data set (multiple loci and multiple samples), a principal coordinate analysis (PCA) was performed using GenAlEx Version 6.3 [40] on a covariance matrix (with data standardization) of pair wise population PhiPT values. A PhiPT measure suppresses intra-population variance and simply calculates population differentiation based on the genotypic variance. PCA reduces the allele frequency information into a small number of synthetic variables and provides a graphical representation of genetic distance.

### Gene flow analysis

To obtain an estimate of the magnitude and direction of contemporary gene flow among populations, a genetic assignment method was used with BayesAss.ver.1.3 [23]. This package uses a fully Bayesian Markov chain Monte Carlo (MCMC) resampling method to estimate the posterior probability distribution of the proportion of migrants from one population to another. The amount of dispersal is estimated by *m*, where *m* is the proportion of each population having immigrant ancestry and where first-generation immigrants or offspring of two immigrant parents will be considered as having full immigrant ancestry and offspring of one immigrant and one native parent will be considered as having half immigrant ancestry. It also calculates a confidence interval for results that would be returned from uninformative data, typically those that do not contain sufficient variation to estimate dispersal with high confidence [23,48]. A run with burn-in-period of 250,000 iterations was performed and was followed by a run length of 500,000 MCMC with a sampling frequency of 2000 generations, using the default parameter setting in the BayesAss program.

## Authors’ contributions

HL, GJ, and MAW coordinated the study. TLR and GJ collected samples and prepared DNA samples for genetic analyses. TLR, HL, MSI carried out genotyping of phylloxera samples. MSI, MAW and HL analyzed results and wrote the paper. All authors read and approved the final manuscript.

## Supplementary Material

Additional file 1: Table S1 Distributions of multilocus genotypes of grape phylloxera among the populations from four vineyard-sites in Napa (Oakville) and Yolo (Woodland) counties, California. Click here for file

## References

[B1] Granett J Walker MA Kocsis L Omer AD Biology and management of grape phylloxera Annu Rev Entomol 2001 14 387 412 10.1146/annurev.ento.46.1.387 11112174

[B2] Wapshere AJ Helm KF Phylloxera and *Vitis*: an experimentally testable co-evolutionary hypothesis Amer J Enol Viticult 1987 14 16 22

[B3] Granett J Fong G Walker A Lin H De Benedictis J Weber E California grape phylloxera more variable than expected Calif Agric 1996 14 9 13

[B4] Granett J Bisabri-Ershadi B Carey J Life tables of phylloxera on resistant and susceptible grape rootstocks Entomol Exper Applic 1983 14 13 19 10.1111/j.1570-7458.1983.tb03284.x

[B5] Granett J Limper P Lider LA phylloxera (*Daktulosphaira vitifoliae*) (Homoptera: Phylloxeridae) biotypes in California J Econ Entomol 1985 14 1463 1467

[B6] Forneck A Huber L (A)sexual reproduction – a review of life cycles of grape phylloxera, *Daktulosphaira vitifoliae* Entomol Exper Applic 2009 14 1 10 10.1111/j.1570-7458.2008.00811.x

[B7] Davidson WM Nougaret RL The grape phylloxera in California 1921 Washington, DC, USA: United States Department of Agriculture; Bulletin 903

[B8] Vorwerk S Forneck A Reproductive mode of grape phylloxera (*Daktulosphaira vitifoliae*, Homoptera: Phylloxeridae) in Europe: molecular evidence for predominantly asexual populations and a lack of gene flow between them Genome 2006 14 678 687 10.1139/G06-028 16936847

[B9] Corrie AM Crozier RH Van Heeswijck R Hoffmann AA Clonal reproduction and population genetic structure of grape phylloxera, *Daktulosphaira vitifoliae*, in Australia Heredity 2002 14 203 211 10.1038/sj.hdy.6800028 11920122

[B10] Corrie AM Hoffmann AA Fine-scale genetic structure of grape phylloxera from the roots and leaves of *Vitis* Heredity 2004 14 118 127 10.1038/sj.hdy.6800393 14679391

[B11] Forneck A Walker MA Blaich R Genetic structure of an introduced pest, grape phylloxera (*Daktulosphaira vitifoliae* Fitch), in Europe Genome 2000 14 669 678 10984180

[B12] Lin H Walker MA Hu R Granett J New simple sequence repeat loci for the study of grape phylloxera (*Daktulosphaira vitifoliae*) genetics and host adaptation Amer J Enol Viticult 2006 14 33 40

[B13] Downie DA Locating the sources of an invasive pest, grape phylloxera, using a mitochondrial DNA gene genealogy Mol Ecol 2002 14 2013 2026 10.1046/j.1365-294X.2002.01584.x 12296945

[B14] Fong G Walker MA Granett J RAPD assessment of California phylloxera diversity Mol Ecol 1995 14 459 464 10.1111/j.1365-294X.1995.tb00239.x

[B15] Downie DA Granett J Fisher JR Distribution and abundance of leaf galling and foliar sexual morphs of grape phylloxera (Hemiptera: Phylloxeridae) and *Vitis* species in the central and eastern United States Environ Entomol 2000 14 979 986 10.1603/0046-225X-29.5.979

[B16] Lin H Downie DA Walker MA Granett J English-Loeb G Genetic structure in native populations of grape phylloxera (Homoptera: Phylloxeridae) Annal Entomol S Amer 1999 14 376 381

[B17] Sunnucks P De Barro PJ Lushai G Maclean N Hales D Genetic structure of an aphid studied using microsatellites: cyclic parthenogenesis, differentiated lineages and host specialization Mol Ecol 1997 14 1059 1073 10.1046/j.1365-294X.1997.00280.x 9394464

[B18] Hales DH Tomiuk J Wöhrmann K Sunnucks P Evolutionary and genetic aspects of aphid biology: a review Eur J Entomol 1997 14 1 55

[B19] Sunnucks P England PR Taylor AC Hales DF Microsatellite and chromosome evolution of parthenogenetic *Sitobion* aphids in Australia Genetics 1996 14 747 756 8889535 10.1093/genetics/144.2.747 PMC1207565

[B20] Fuller SJ Chavigny P Lapchin L Vanlerberghe-Masutti F Variation in clonal diversity in glasshouse infestations of the aphid, *Aphis gossypii* Glover in southern France Mol Ecol 1999 14 1867 1877 10.1046/j.1365-294x.1999.00782.x 10620230

[B21] Simon JC Baumann S Sunnucks P Hebert PD Pierre JS Le Gallic JF Dedryver CA Reproductive mode and population genetic structure of the cereal aphid *Sitobion avenae* studied using phenotypic and microsatellite markers Mol Ecol 1999 14 531 545 10.1046/j.1365-294x.1999.00583.x 10327655

[B22] Wilson AC Sunnucks P Hales DF Microevolution, low clonal diversity and genetic affinities of parthenogenetic *sitobion* aphids in New Zealand Mol Ecol 1999 14 1655 1666 10.1046/j.1365-294x.1999.00751.x 10583829

[B23] Wilson GA Rannala B Bayesian inference of recent migration rates using multilocus genotypes Genetics 2003 14 1177 1191 12663554 10.1093/genetics/163.3.1177 PMC1462502

[B24] Halkett F Simon JC Balloux F Tackling the population genetics of clonal and partially clonal organisms Trends Ecol Evol 2005 14 194 201 10.1016/j.tree.2005.01.001 16701368

[B25] Ivens AB Kronauer DJ Pen I Weissing FJ Boomsma JJ Reproduction and dispersal in an ant-associated root aphid community Mol Ecol 2012 14 4257 4269 10.1111/j.1365-294X.2012.05701.x 22804757

[B26] de Meeus T Balloux F Clonal reproduction and linkage disequilibrium in diploids: a simulation study Infect Genet Evol 2004 14 345 351 10.1016/j.meegid.2004.05.002 15374532

[B27] Balloux F Heterozygote excess in small populations and the heterozygote-excess effective population size Evolution 2004 14 1891 1900 15521449 10.1111/j.0014-3820.2004.tb00477.x

[B28] Stellwaag-Kittler F Das Auftreten der geflügelten Reblaus Der Deutsche Weinbau, 24 1954 14 737 738

[B29] Downie DA Granett J A life cycle variation in grape phylloxera Southwest Entomol 1998 14 11 16

[B30] Downie DA Effects of short-term spontaneous mutation accumulation for life history traits in grape phylloxera, *Daktulosphaira vitifoliae* Genetica 2003 14 237 251 14686603 10.1023/b:gene.0000003610.73205.c7

[B31] Kimberling DN Price PW Competition, leaf morphology, and host clone effects on leaf-galling grape phylloxera (Homoptera: Phylloxeridae) Environ Entomol 1996 14 1147 1153

[B32] Kocsis L Granett J Walker MA Lin H Omer AD Grape phylloxera populations adapted to *Vitis berlandieri* x *V.riparia* rootstocks Amer J Enol Viticult 1999 14 101 106

[B33] Forneck A Walker MA Blaich R An in vitro assessment of phylloxera (*Daktulosphaira vitifoliae* Fitch) life cycle J Appl Entomol 2001 14 1052 1054

[B34] Corrie AM van Heeswijck R Hoffmann AA Evidence for host-associated clones of grape phylloxera *Daktulosphaira vitifoliae* (Hemiptera: Phylloxeridae) in Australia Bull Entomol Res 2003 14 193 201 12762861 10.1079/BER2003232

[B35] Downie DA Patterns of genetic variation in native grape phylloxera on two sympatric host species Mol Ecol 2000 14 505 514 10.1046/j.1365-294x.2000.00684.x 10792695

[B36] Qing-Hua S Ying-Chun C Hai-Bo W Downie DA Heng Z Origin and genetic diversity of grape phylloxera in China Acta Entomol Sin 2009 14 885 894

[B37] Lin H Walker MA Extraction of DNA from a single egg of grape phylloxera (*Daktulosphaira vitifoliae* Fitch) for use in RAPD testing Vitis 1996 14 87 89

[B38] Stenberg P Lundmark M Saura A MLGsim: a program for detecting clones using a simulation approach Mol Ecol Notes 2003 14 329 331

[B39] Ivey CT Richards JH Genetic diversity of everglades sawgrass, *Cladium jamaicense* (Cyperaceae) Internat J Plant Sci 2001 14 817 825 10.1086/320775

[B40] Peakall R Smouse P GENALEX 6: genetic analysis in excel. Population genetic software for teaching and research Mol Ecol Notes 2006 14 288 295 10.1111/j.1471-8286.2005.01155.x PMC3463245 22820204

[B41] Nei M Estimation of average heterozygosity and genetic distance from a small number of individuals Genetics 1978 14 583 590 17248844 10.1093/genetics/89.3.583 PMC1213855

[B42] Goudet J FSTAT (Version 1.2): a computer program to calculate F-statistics J Hered 1995 14 485 486

[B43] Wright S Evolution and the genetics of populations. Variability within and among natural populations 1978 Chicago: University of Chicago Press

[B44] Raymond M Rousset F GENEPOP (version 1.2): population genetics software for exact tests and ecumenicism J Hered 1995 14 248 249

[B45] Guo SW Thompson EA Performing the exact test of Hardy-Weinberg proportion for multiple alleles Biometrics 1992 14 361 372 10.2307/2532296 1637966

[B46] Nei M Tajima F Tateno Y Accuracy of estimated phylogenetic trees from molecular data. II. Gene frequency data J Mol Evol 1983 14 153 170 10.1007/BF02300753 6571220

[B47] Tamura K Dudley J Nei M Kumar S MEGA4: molecular evolutionary genetics analysis (MEGA) software version 4.0 Mol Biol and Evol 2007 14 1596 1599 10.1093/molbev/msm092 17488738

[B48] Pearse DE Crandall KA Beyond *F*_ST_: analysis of population genetic data for conservation Conserv Genet 2004 14 585 602 10.1007/s10592-003-1863-4

