# Supplementary material for: Reproductive mode and fine-scale population genetic structure of grape phylloxera (Daktulosphaira vitifoliae) in a viticultural area in California
Source: BMC Genet. 2013 Dec 24;14:123. doi: 10.1186/1471-2156-14-123 (PMC3890642; doi:10.1186/1471-2156-14-123)
Supplement: Additional file 1: Table S1 — Distributions of multilocus genotypes of grape phylloxera among the populations from four vineyard-sites in Napa (Oakville) and Yolo (Woodland) counties, California. [file 1471-2156-14-123-S1.doc]

**Additional file 1: Table S1.** Distributions of multilocus genotypes of grape phylloxera among the populations from four study sites in Napa (Oakville) and Yolo (Woodland) counties, California.
